# Vitamin D and skin disorders: bridging molecular insights to clinical innovations

**DOI:** 10.1186/s10020-025-01311-5

**Published:** 2025-07-18

**Authors:** Qing Li, Hung Chan

**Affiliations:** 1 Dermatology Hospital, Southern Medical University, Guangzhou, China; 2 Department of Dermatology, University of California San Diego, San Diego, La Jolla, CA USA

**Keywords:** Vitamin D deficiency, Vitamin D receptor, Skin diseases

## Abstract

Growing evidence demonstrates that the immunoregulatory properties of vitamin D are primarily mediated by its active hormonal form, 1,25-dihydroxyvitamin D3 (calcitriol). This secosteroid modulates immune homeostasis through three principal mechanisms: (1) strengthening antimicrobial defense via innate immune potentiation, (2) downregulating pathological inflammatory cascades, and (3) fine-tuning adaptive immunity through lymphocyte differentiation control. Clinically, serum concentrations of the inactive precursor, 25-hydroxyvitamin D3 (25(OH)D3), exhibit an inverse correlation with systemic immune activation and the prevalence/severity of dermatological conditions, including atopic dermatitis, psoriasis, and systemic sclerosis. Suboptimal 25(OH)D3 levels are thus recognized as a modifiable risk factor for such disorders, with vitamin D3 supplementation demonstrating therapeutic potential in improving clinical outcomes. Furthermore, prolonged vitamin D3 supplementation may reduce disease incidence across a spectrum of dermatopathologies. This review synthesizes contemporary mechanistic and clinical insights into vitamin D’s immunoregulatory role in cutaneous diseases. To optimize therapeutic efficacy, future clinical trials should incorporate analysis of vitamin D receptor (VDR) polymorphisms as a predictive biomarker in vitamin D3 intervention strategies.

## Introduction

Of interest are the considerable and increasing problems caused by dermatological conditions, which impact almost half of the global population. They are a primary reason for patients to present themselves for treatment in general practice and are classified as the fourth foremost cause of non-fatal health burden (Richard et al. 2022).Dermatoses exert substantial adverse effects on individuals'daily functioning and occupational performance, often leading to stigma and exacerbating psychological distress (2023).

Vitamin D3 deficiency has been studied in connection with the progression of dermatological diseases for over a decade. The focus has shifted from its original role in calcium and phosphorus homeostasis in skeletal health to its steroid-like properties. However, consensus on its precise role remains elusive. Significant attention has been given to the potential involvement of vitamin D3 metabolism, particularly its dysregulation in dermatological diseases (Cui A et al. 2023; Kechichian and Ezzedine 2018). Emphasizing the importance of specific molecular processes associated with vitamin D3 in host health preservation is essential, especially for physicians who practice in the field of patient care. Interventions intended to rebalance vitamin D3 homeostasis present an effective method of prevention and therapy for skin ailments. In this review, the correlation between a lack of vitamin D3 and diverse dermatoses is discussed first. We then outline the possible therapeutic consequences of vitamin D3 treatment, along with the mechanisms involved. This is followed by consideration of how the vitamin D3 receptor is likely to impact the results of clinical trials.

## Vitamin D3 metabolism

**Fig. 1:**
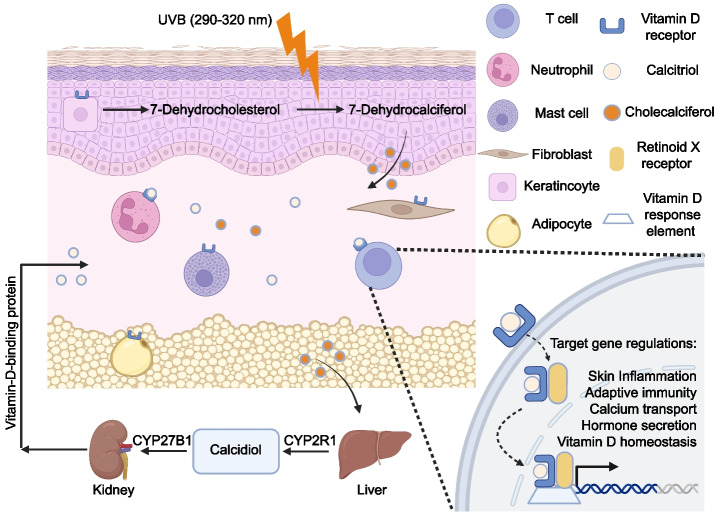
Vitamin D metabolic pathway and pleiotropic impacts on the skin. Vitamin D primarily originates from vitamin D3 synthesized in the skin upon exposure to ultraviolet B (UVB) radiation from sunlight. The kidneys produce the majority of circulating 1,25-dihydroxyvitamin D3 (1,25(OH)2D3), which functions endocrinologically. Additionally, extra-renal synthesis of 1,25(OH)2D3 by local 1α-hydroxylase (CYP27B1) permits localized functions. 1,25(OH)2D3 exerts its effects by binding to the vitamin D receptor (VDR) present on various cell types, including T cells, neutrophils, mast cells, fibroblasts, keratinocytes, and adipocytes. This binding induces the VDR to heterodimerize with the retinoid X receptor (RXR). The ligand-bound VDR-RXR complex then attaches to vitamin D response elements (VDRE) in the promoter regions of target genes, regulating gene expression related to skin inflammation, vitamin D homeostasis, adaptive immunity, calcium transport, and hormone secretion

The skin serves as the principal organ for vitamin D synthesis, with approximately 80% of endogenous vitamin D production occurring via ultraviolet B (UVB) radiation–dependent conversion of 7-dehydrocholesterol (7-DHC) to pre-vitamin D3 in epidermal keratinocytes. This photochemical reaction is subsequently followed by thermal isomerization of pre-vitamin D3 to vitamin D3 (cholecalciferol). Following hepatic hydroxylation to 25-hydroxyvitamin D3 (25(OH)D3) and renal activation to 1,25-dihydroxyvitamin D3 (1,25(OH)2D3; calcitriol), the hormonally active metabolite binds to the vitamin D receptor (VDR) in cutaneous tissues, regulating multiple physiological processes, including cellular differentiation, proliferation, and immune modulation (Fig. 1), including proliferation, differentiation, apoptosis, barrier integrity, and immunoregulatory functions (Bikle 2012).

Genetic polymorphisms in key vitamin D metabolic enzymes (e.g., CYP2R1, CYP27B1) modulate serum 25-hydroxyvitamin D (25OHD3) responsiveness to exogenous sources, including dietary supplementation and cutaneous UVB-induced synthesis. These variations underlie interindividual variability in 25OHD3 bioavailability, with implications for personalized vitamin D supplementation strategies (Gospodarska et al. 2023). The enzyme 1α-hydroxylase (1α-OHase), encoded by the CYP27B1 gene, catalyzes the bioactivation of 25-hydroxyvitamin D3 (25(OH)D3) to its hormonally active form, 1α,25-dihydroxyvitamin D3 (1,25(OH)2D3; calcitriol), as depicted in Fig. 1. This reaction occurs predominantly in renal proximal tubules and extrarenal tissues (e.g., keratinocytes, immune cells), with CYP27B1 expression tightly regulated by parathyroid hormone, calcium, and phosphate levels. The resultant calcitriol binds to the VDR, triggering pleiotropic genomic and non-genomic responses that regulate calcium homeostasis, cellular differentiation, and immune modulation (Deeb et al. 2007). Genetic polymorphisms in the VDR gene can induce functional impairment of the receptor, compromising its ligand-binding affinity or transcriptional activity. Such VDR variants have been implicated in the pathogenesis of cutaneous disorders through dysregulation of epidermal keratinocyte proliferation and differentiation—processes critical to skin barrier integrity and homeostatic regulation (Umar et al. 2018). Ligand-dependent activation of the VDR initiates canonical signaling via heterodimerization with the retinoid X receptor (RXR). The heterodimeric VDR-RXR complex binds cognate vitamin D response elements (VDREs) in target gene promoters, initiating transcriptional activation of immunomodulatory and cell fate-determining genes. These downstream effectors orchestrate critical pathophysiological processes such as inflammatory cascades, cell cycle progression, and lineage-specific differentiation programs (Deeb et al. 2007).

## Vitamin D3 in dermatologic conditions

### Atopic dermatitis

Atopic dermatitis (AD), a chronic, relapsing inflammatory dermatosis characterized by a complex interplay of genetic, immunological, and environmental factors, presents clinically with hallmark features including chronic pruritus, marked xerosis, and erythematous eczematous plaques. Its pathogenesis involves dysregulation of skin barrier function (e.g., filaggrin mutations), aberrant Th2/Th22 immune polarization and microbial dysbiosis, contributing to its persistent and often refractory nature (Fang et al. 2021; Hidaka et al. 2017; Langan et al. 2020; Khan 2025). Robust observational evidence demonstrates an inverse correlation between the clinical severity of AD and serum concentrations of 25-hydroxyvitamin D3 (25(OH)D3), suggesting that hypovitaminosis D may exacerbate cutaneous inflammation and barrier dysfunction in AD pathogenesis (Ding et al. 2024; Ibrahim et al. 2020; Barlianto et al. 2022; Dogru 2018)(Table 1). Decreased levels of maternal 25(OH)D_3_ were associated with an increased susceptibility to infantile eczema. A positive correlation was observed between maternal 25(OH)D_3_ levels and Foxp3 gene expression in cord blood (Ding et al. 2024).

**Table 1:** Clinical interventional evidence indicating the impact of vitamin D supplementation on atopic dermatitis and outcomes

Sample size	Country	Intervention (vitamin D vs placebo)	Duration of follow-up	Primary outcomes	Results	References
1694	UK	1000 IU daily	12–48 months	neonatal whole-body bone mineral content & prevalence of atopic eczema	Antenatal vitamin D supplementation is potentially beneficial	(Dogru, M. 2018)
86	Egypt	1600 IU daily	12 weeks	mean Eczema Area and Severity Index (EASI) score	Vitamin D3 improves the clinical outcomes in severe atopic dermatitis	(Kang, S.W et al. 2012)
81	Iran	1000 IU daily	2 months	SCORing Atopic Dermatitis (SCORAD)	Vitamin D3 reduces the severity of AD in infants	(Hori, S 2003)
45	Canada	2000 IU daily	3 months	SCORAD severity	VD supplementation doesn't significantly improve disease severity	(Rudra D et al. 2012)
65	Mexico	3500 IU daily	3 months	SCORAD severity	Achieving serum levels of 25(OH)D greater than 20 ng/ml alongside standard therapy is sufficient to achieve a reduction in severity (SCORAD) in patients with AD	(Chaudhry A et al. 2009)
24	Thailand	2000 IU daily	2–4 weeks	SCORAD severity	Oral vitamin D supplement reduces skin colonization of S. aureus and improved the outcomes of aptoic dermatitis	(Lu R et al 2023)
107	Mongolia	1000 IU daily	one month	Eczema Area and Severity Index (EASI) and Investigator's Global Assessment (IGA)	Vitamin D supplementation improves winter-related AD	(Liu Y 2007)
60	United States	4000 IU daily	21 days	levels of 25-hydroxyvitamin D (25OHD), cathelicidin,	Oral cholecalciferol is no significant change in skin cathelicidin, HBD-3, IL13, or EASI scores	(Lu R et al 2023)
				HBD-3, IL-13, and Eczema Area and Severity Index (EASI) and Rajka-Langeland score		
22	Chile	8000 IU/week for 2–5.9 years; 12,000 IU/week for 6–11.9 years; 16,000 IU/week for 12–18 years	6 weeks	SCORAD severity, levels of 25-hydroxyvitamin D (25OHD) in blood	Oral VD3 supplementation improves AD severity	Varricchi G et al. 2018
25	Chile	8000 IU/week for 2–5.9 years; 12 000 IU/week for 6–11.9 years; 16 000 IU/week for 12–18 years	6 weeks	Circulating myeloid, plasmacytoid dendritic cells,SCORAD severity	Oral VD supplementation reduces expression of surface bound IgE on plasmacytoid dendritic cells in children with AD, but no significant change in SCORAD	Chauss D et al. 2022

Mechanistic studies reveal that 1,25-dihydroxyvitamin D3 (1,25(OH)2D3) upregulates Foxp3 transcription by binding to VDREs within the intronic conserved non-coding sequence (CNS) spanning positions + 1714 to + 2554 of the human Foxp3 locus. As a master transcription factor, Foxp3 drives the immunosuppressive function of natural regulatory T cells (Tregs) — characterized by their CD4^+^CD25^+^phenotype — by modulating signaling pathways critical for Treg differentiation, stability, and suppressive activity (Kang et al. 2012; Hori et al. 2003). Calcifediol not only upregulates Foxp3 expression but also inhibits STAT3 phosphorylation, thereby attenuating Th17-mediated inflammation (Rudra et al. 2012; Chaudhry et al. 2009). This immunomodulatory effect suppresses production of key Th2-associated pro-inflammatory cytokines—including IL-4, IL-13, IL-31, IL-33, CCL17, and thymic stromal lymphopoietin (TSLP)—culminating in marked attenuation of clinical features (erythema, edema), reduced systemic chemokine levels, and diminished dermal infiltration of inflammatory lymphocytes, particularly CD4 + T cells and eosinophils, in a 2,4-dinitrochlorobenzene (DNCB)-induced murine model of atopic dermatitis (Lu et al. 2023; Liu et al. 2007; Varricchi et al. 2018).

Calcifediol (25-hydroxyvitamin D3) engages with plasma complement proteins to amplify complement system activation, thereby modulating Th1-mediated immunity through upregulation of VDR and 1α-hydroxylase (CYP27B1) expression. This primes T cells for vitamin D responsiveness, promoting a phenotypic shift from pro-inflammatory IFN-γ^+^ Th1 effector cells toward immunosuppressive IL-10^+^ regulatory T cells. Concurrently, this immunologic reprogramming is driven by epigenetic remodeling in CD4^+^ T cells, including super-enhancer formation and recruitment of transcription factors (e.g., c-JUN, STAT3, BACH2) that stabilize the immunosuppressive transcriptional program (Chauss et al. 2022; Liszewski et al. 2013). Beyond its effects on cytokine stimulation, vitamin D also modulates the surface markers of immune cells.

Calcifediol prompts terminally differentiated monocyte-derived dendritic cells (MonoDCs) to upregulate both surface and soluble CD14, influencing their phenotype and ability to drive IL-4^+^ Th2 responses (Brulefert et al. 2021). Furthermore, calcifediol antagonizes the binding of the transcription factor PU.1 (SPI1) to the FCER1A promoter, suppressing expression of the high-affinity IgE receptor FcεRI and surface-bound immunoglobulin E (IgE) on Langerhans cells and plasmacytoid dendritic cells (pDCs). This downregulation attenuates IgE-mediated hypersensitivity responses and promotes restoration of epidermal barrier integrity through diminished inflammatory signaling and enhanced keratinocyte differentiation (Lu et al. 2023; Ahmed et al. 2020). This reduction impairs the IgE-mediated allergen signalling pathway in patients with atopic dermatitis (Cristi et al. 2019). While these findings provide mechanistic insights, they are primarily derived from in vitro models, highlighting the need for in vivo studies to elucidate the spatiotemporal dynamics of multicellular and microenvironmental crosstalk critical for mechanistic validation and translational relevance.

### Psoriasis

Psoriasis is classified as a chronic, immune-mediated inflammatory dermatosis marked by dysregulated crosstalk between innate and adaptive immunity, involving autoinflammatory cytokine cascades (e.g., IL-23/IL-17 axis) and autoimmune T-cell activation. Epidemiological data from the most recent World Health Organization report indicate a rising global prevalence, affecting 1.5%–5% of populations in high-income countries, with increasing incidence linked to urbanization, environmental triggers, and genetic susceptibility (Tiuca et al. 2023; Sun et al. 2025; Xiong and Yu 2025). Psoriasis pathogenesis emerges from the convergence of genetic susceptibility (e.g., HLA-C06:02, IL23R variants), environmental triggers (e.g., trauma, infections), and dysregulated immune activation, particularly via the IL-23/Th17 axis. This interplay drives a pathogenic cascade characterized by TNF-α/IL-17/IL-22–mediated keratinocyte hyperproliferation, aberrant differentiation, and epidermal barrier disruption, perpetuating a self-reinforcing inflammatory loop central to psoriatic plaque formation (Boehncke and Schon 2015; Wu et al. 2023; Emmanuel et al. 2025). The expression of VDRs on keratinocytes and immune cells—including T lymphocytes, B lymphocytes, and natural killer cells—implies that vitamin D insufficiency or VDR polymorphisms may disrupt immunomodulation in inflammatory milieus. Such perturbations can exacerbate keratinocyte hyperproliferation, aberrant differentiation, and immune dysregulation, hallmarks of psoriatic pathophysiology. This mechanistic link underscores the therapeutic potential of vitamin D analogs (e.g., calcipotriol) and highlights the need to address vitamin D deficiency as an adjunct strategy in psoriasis management (Solak et al. 2016; Hambly and Kirby 2017; Yang et al. 2025a; Jimenez-Sanchez et al. 2025; Yi et al. 2025).

An inverse correlation has been observed between serum 25-hydroxyvitamin D (25(OH)D) concentrations and clinical metrics of psoriasis severity, including disease duration, Psoriasis Area and Severity Index (PASI), and systemic inflammatory markers (e.g., erythrocyte sedimentation rate [ESR]). Furthermore, advancing age and female sex are independent demographic factors associated with significantly lower serum 25(OH)D levels, even after adjustment for confounding variables such as UV exposure and dietary intake (Mohta and Nyati 2022). To address vitamin D insufficiency and its clinical sequelae in psoriasis, therapeutic strategies utilizing vitamin D analogs have been investigated. In patients with psoriasis vulgaris, combination therapy with calcipotriol (50 µg/g; a vitamin D3 analog) and betamethasone dipropionate (0.5 mg/g; a high-potency corticosteroid) foam demonstrates marked reductions in histopathological and sonographic markers of disease activity. Specifically, this regimen significantly decreases total epidermal-dermal thickness and sonographically quantified echo-poor band thickness—a surrogate for superficial dermal inflammation—alongside improvements in targeted Psoriasis Area and Severity Index (t-PASI) scores. These outcomes are attributed to calcipotriol’s dual anti-inflammatory and antiproliferative effects via VDR-mediated keratinocyte modulation, synergizing with betamethasone’s immunosuppressive action to restore epidermal homeostasis (Tada et al. 2021; Heim et al. 2022). Clinical evidence from a prospective cohort study enrolling 105 patients with 177 psoriatic lesions treated with once-daily calcipotriol/betamethasone dipropionate (Cal/BD) foam over a 4-week period demonstrated statistically significant reductions in lesion surface area and pruritus severity (*p* < 0.01), with no treatment-related adverse events reported, validating its short-term tolerability profile (Wu et al. 2019).

In psoriasis, dendritic cells initiate an autoimmune response by expressing major histocompatibility complex I in response to inflammatory mediators or microbial products. These cells then migrate to T cell-enriched areas, where they interact with T cell surface markers and secrete cytokines that drive T cell differentiation (Cabeza-Cabrerizo et al. 2021). This process activates T cells, leading to their differentiation into various T helper cell subsets, including Th1 and Th17 (Rendon and Schakel 2019; Dopytalska et al. 2021). The expansion of T helper cells, particularly Th17, further stimulates the production of pro-inflammatory cytokines such as TNFα and IL-17A, leading to keratinocyte hyperproliferation. Stressed keratinocytes then trigger IL-1β production, recruiting neutrophils to amplify the inflammatory response through the formation of neutrophil extracellular traps (NETs)—key elements in the pathogenesis and progression of psoriasis complications (Capriotti et al. 2022; Meng et al. 2022). The migrated neutrophil subsequently provokes the production of IL-17 and IFN-γ by T cells (Rodriguez-Rosales et al. 2021), creating a vicious cycle that drives the progression of psoriasis.

**Fig. 2:**
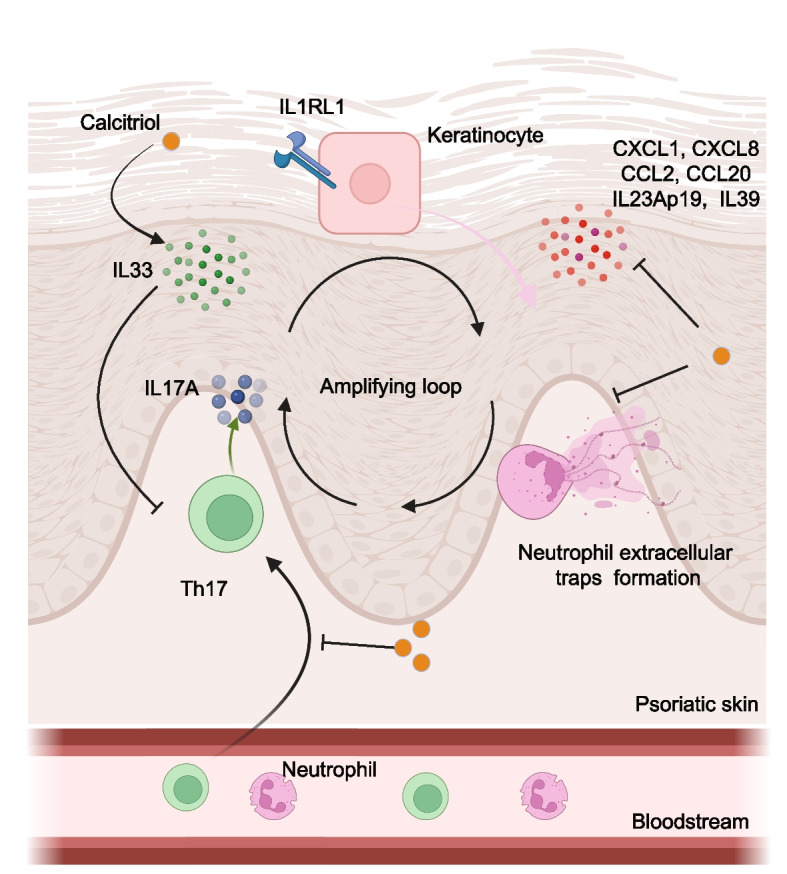
Summarized molecular mechanism of calcitriol in psoriasis. The molecular mechanism of calcitriol in psoriasis involves its action on psoriatic keratinocytes, where it stimulates the production of the anti-inflammatory cytokine IL-33 and upregulates IL1RL1 expression. IL-33, induced by calcitriol, subsequently inhibits IL-17A production by Th17 cells. Additionally, IL-33 suppresses a range of pro-inflammatory cytokines and chemotactic cytokines while also inhibiting the formation of neutrophil extracellular traps. The inhibition of chemotactic cytokines prevents the migration of Th17 cells from the bloodstream to psoriatic skin. IL17A, interleukin 17 A; CCL2, chemokine (C–C motif) ligand 2; CCL20, chemokine (C–C motif) ligand 20; CXCL1, chemokine (C-X-C motif) ligand 1; CXCL8, interleukin 8; IL1RL1/ST2, interleukin 1 receptor-like 1; IL23Ap19, interleukin 23, alpha subunit p19; IL39, interleukin 39; Th17, type 17 T helper cells

The mechanistic basis of Cal/BD foam in psoriasis therapy involves multimodal immunomodulation targeting T cell subsets, keratinocytes, and neutrophils, as detailed in Fig. 2. Cal/BD treatment suppresses epidermal and dermal infiltration of pathogenic CD8 + T cell populations, including IFN-γ–producing CD8 + T cells (CD8 + IFN-γ +) and tissue-resident memory T cells (TRM; CD8 + CD103 +), while concurrently reducing myeloperoxidase-positive (MPO +) neutrophils—key contributors to IL-17–driven inflammation. This dual action disrupts the IL-23/Th17 axis, attenuates keratinocyte hyperproliferation, and resolves plaque-associated neutrophilic micro abscesses, synergizing calcipotriol’s VDR–mediated genomic regulation with betamethasone’s glucocorticoid receptor (GR)–dependent anti-inflammatory effects (Heim et al. 2022; Thome and Farber 2015; Hidalgo et al. 2010). Notably, Cal/BD foam exhibits no significant modulatory effect on CD8^+^IL-17^+^ T cells or natural killer cell populations (Heim et al. 2022). The observed attenuation of CD8 + T cell trafficking is mediated by a rebalancing of immunosuppressive regulatory T cell subsets (CD8^+^ T_reg_ and CD4^+^ T_reg_) and proinflammatory CCR6 + γδT17 cells within draining lymph nodes, thereby re-establishing immune homeostasis (Satake et al. 2019). Skin-draining lymph nodes (SDLNs) serve as critical secondary lymphoid organs for antigen-presentation and priming of naïve T cells against cutaneous antigens. Following activation, effector T cells traffic from SDLNs to the skin via circulatory routes, while tissue-resident memory T cells (TRM) in the epidermis and dermis can recirculate back to SDLNs, thereby priming systemic immunity against recurrent antigenic challenges and amplifying cross-talk between cutaneous and systemic immune compartments (Catron et al. 2006; Teijeira et al. 2017; 2024).Although CB treatment clinically improves skin lesions, it does not eliminate all TRM in the basement membrane zone, leaving the possibility of future psoriasis episodes (Thome and Farber 2015; Kurihara et al. 2022). Further research is needed to understand the mechanisms by which TRM escape CB treatment, leading to subsequent episodes of relapse.

Vitamin D3 transcriptionally suppresses the expression of pathogenic pro-inflammatory cytokines, including IL-23A (p19) and IL-39 in psoriatic keratinocytes, thereby attenuating IL-23/Th17 axis activation and disrupting autocrine-paracrine inflammatory loops central to psoriasis pathogenesis (Tachibana et al. 2021). Besides, 1,25(OH)_2_D_3_ effectively stimulates expression of IL-33 and its receptor suppression of tumorigenicity 2 (ST2;IL1RL1) mRNAs in keratinocytes. Functioning as both a nuclear chromatin-associated regulator and a soluble alarmin, IL-33 plays dual roles in maintaining cutaneous tissue homeostasis, promoting wound repair via re-epithelialization and extracellular matrix remodelling, and orchestrating type 2 immune responses through activation of CD4 + Th2 lymphocytes, mast cells, and group 2 innate lymphoid cells (ILC2s). Upon tissue injury or barrier disruption, IL-33 is released as a damage-associated molecular pattern (DAMP), driving inflammatory cascades linked to atopic and fibrotic skin disorders (Nile et al. 2010; 2025; Yang et al. 2025b). IL-33 exerts immunomodulatory effects by suppressing IL-17A expression in CD4^+^T cells isolated from psoriasis patients, likely through inhibition of STAT3-dependent IL17A transcription. In a murine model of psoriatic inflammation, subcutaneous IL-33 administration attenuates epidermal hyperplasia and dermal inflammation, concomitant with downregulation of pro-inflammatory cytokines TNF-α and IL-23 in lesional skin. This therapeutic effect correlates with a reduced frequency of IL-17A^+^CD4^+^Th17 cells in SDLNs, suggesting systemic modulation of the IL-23/Th17 axis. Mechanistically, IL-33 may impair Th17 differentiation via suppression of RORγt (RORC) and STAT3 signalling while promoting Treg expansion, rebalancing immune homeostasis in psoriasis (Chen et al. 2020).

Interestingly, there is a distinct pattern in the expression of IL-33 and ST2 in psoriasis. Interleukin-33 (IL-33) is markedly elevated in lesional skin and serum of patients with moderate-to-severe plaque psoriasis (Chen et al. 2020). yet its receptor, suppression of tumorigenicity 2 (ST2; IL1RL1), is transcriptionally downregulated in psoriatic plaques compared to perilesional and healthy skin (Wierzbicka et al. 2021). This paradox—wherein IL-33 acts as an epithelial-derived alarmin to activate innate immune cells (e.g., mast cells, ILC2s) and adaptive Th2 responses—suggests context-dependent roles in both initiating and resolving inflammation. Notably, vitamin D3 treatment restores ST2 expression in psoriatic keratinocytes, implicating dysregulated IL-33/ST2 signaling as a therapeutically targetable node. By rescuing receptor sensitivity, vitamin D3 may recalibrate IL-33’s dual immunomodulatory functions, attenuating pathogenic inflammation while preserving its homeostatic tissue-repair roles. In neutrophils, calcitriol has been shown not only to prevent the formation of NETs with a daily intake of 1000 IU of vitamin D3 (Basyreva et al. 2023), but also to reduce the pro-inflammatory process by inhibiting neutrophil migration (Takahashi et al. 2002; Anderson et al. 2020) through the decreased production of CXCL1 and CCL20 (Takei-Taniguchi et al. 2012; Xin et al. 2021).

Genetic polymorphisms in the VDR, particularly the TaaI/Cdx-2 (rs11568820) single-nucleotide polymorphism (SNP), are associated with susceptibility to psoriasis and other immune-mediated dermatoses (Table 2) (Rucevic et al. 2012). The homozygous GG genotype of this VDR SNP is significantly overrepresented in psoriatic cohorts, correlating with dysregulated serum concentrations of IL-17A and IL-23p19. This genotype-dependent dysregulation likely arises from altered VDR-mediated transcriptional control of IL17A and IL23A loci, implicating VDR polymorphisms as modifiers of IL-23/Th17 axis hyperactivity in psoriasis pathogenesis. Furthermore, these TaaI/Cdx-2 variants have a notable impact on the response to phototherapy in psoriasis patients. Specifically, they modulate the inflammatory response by reducing IL-17 and IL-23 levels following UVB phototherapy, with significant findings observed particularly in the Polish population (Lesiak et al. 2021). Notably, the findings of this study must be interpreted with consideration of its methodological constraints, particularly the modest cohort size, which may limit statistical power and broader generalizability. As a result, the association between the Bsml and FokI genotypes with psoriasis is weak, and no significant association is found with the ApaI genotype. It is intriguing to observe that even within the same country, the distribution of VDR SNPs can vary regionally. In China, a study found that variant alleles of ApaI and BsmI are associated with increased susceptibility to psoriasis vulgaris in East China, while in north-eastern China, only ApaI is identified as a psoriasis-risk locus (Liu et al. 2020). Further research with larger sample sizes and consideration of epigenetic factors is needed to gain more comprehensive insights into these genetic associations.

**Table 2:** Impact of VDR polymorphisms on skin diseases

Gene polymorphism	Variants	Population	Skin diseases	References
FokI	rs2228570	Tunisian	Pemphigus foliaceous	(Anderson J et al. 2020)
		Tunisian	Pemphigus vulgaris	(Takei-Taniguchi R et al. 2012)
		Lithuania	atopic dermatitis	(Xin Y et al. 2021)
		Han Chinese	psoriasis	(Takahashi K et al. 2002)
		Colombian Caribbean	Chronic spontaneous urticaria	(Rucevic I et al. 2012)
TaqI	rs731236	Lithuania	atopic dermatitis	(Xin Y et al. 2021)
		Caucasian	Psoriasis	(Lesiak A et al. 2021)
BsmI	rs1544410	Turks	Atopic dermatitis	(Liu, J et al. 2020)
		Han Chinese	psoriasis	(Takahashi, K et al. 2002)
ApaI	rs7975232	Han Chinese	Systemic Sclerosis	(Goodarzi A et al. 2020)
		Egyptian	Acne vulgaris	(Chen Y et al. 2025)
		Han Chinese	psoriasis	(Takahashi K et al. 2002)
		Saudi Arabian	psoriasis	(Cordain L et al 2002)
Cdx-2	rs11568820	Saudi Arabian	psoriasis vulgaris	(Makrantonaki E et al. 2011)
TaqI	rs731236	Egyptian	Acne vulgaris	(Chen Y et al. 2025)
		Colombian Caribbean	Chronic spontaneous urticaria	(Rucevic I et al. 2012)
Bgll	rs739837	Han Chinese	Systemic Sclerosis	(Goodarzi A et al. 2020)

### Acne vulgaris

Acne represents a chronic inflammatory skin condition affecting hair follicles and sebaceous glands, primarily afflicting individuals in their youth. This dermatological condition significantly compromises patients'quality of life, manifesting in reduced self-esteem, challenges in social interactions, and psychological distress (Goodarzi et al. 2020; Chen et al. 2025). Acne vulgaris stands as the prevailing skin disorder in the Western hemisphere, with an extensive impact on adolescents. Studies indicate its prevalence ranging between 79 and 95% among this demographic group (Cordain et al. 2002). Acne vulgaris arises from a multifactorial pathogenesis involving: (1)sebaceous gland hypersecretion of sebum, driven by androgen-mediated glandular hyperplasia (Makrantonaki et al. 2011); (2) follicular hyperkeratinisation, leading to pilosebaceous duct occlusion (Cunliffe et al. 1976); (3) dysbiotic proliferation of *Cutibacterium acnes* (*C. acnes*), which hydrolyzes sebum triglycerides into pro-inflammatory free fatty acids (Sun et al. 2024); and (4) TLR2/4-dependent activation of innate immunity, triggering a neutrophilic inflammatory cascade that exacerbates tissue damage (Suh and Kwon 2015; Kaplan et al. 2025; Mias et al. 2023).

Observational data reveal a four-fold higher prevalence of 25-hydroxyvitamin D (25(OH)D) deficiency among acne patients compared to healthy controls, with serum 25(OH)D levels inversely correlated with acne severity (Singh et al. 2021). Mechanistically, hypovitaminosis D3 is inversely associated with elevated IL-17 concentrations, suggesting a role for dysregulated Th17-mediated immunity in acne pathogenesis (Abd-Elmaged et al. 2019). Clinically, a 2-month intervention with topical calcipotriol 0.005% cream—a low-calcemic vitamin D analog—demonstrated significant reductions in inflammatory, non-inflammatory, and total acne lesions, alongside marked improvements in both clinician- and patient-reported global assessment scores (Abdel-Wahab et al. 2022). Weekly vitamin D2 supplementation notably prevents the relapse of inflammatory acne lesions at follow-up, with no adverse effects or biochemical changes observed (Ruikchuchit and Juntongjin 2024; Mahran et al. 2024). These findings underscore vitamin D’s dual role in modulating sebocyte homeostasis and curtailing IL-17–driven inflammation, positioning it as a promising adjunctive therapy in acne management.

1,25-dihydroxyvitamin D3 exhibits multifaceted therapeutic potential in acne vulgaris through anti-comedogenic, antioxidant, and immunomodulatory mechanisms. By upregulating key antioxidant enzymes—superoxide dismutase (SOD) and glutathione peroxidase (GSH-Px)—it counteracts oxidative stress, which is characteristically reduced in papulopustular acne lesions (Bowe and Logan 2010; Basak et al. 2001). Concurrently, vitamin D3 enhances the synthesis of antimicrobial peptides such as cathelicidins, directly targeting *Cutibacterium acnes* colonization and mitigating microbial-driven inflammation (Xi et al. 2024; O'Neill et al. 2022). Interestingly, the antimicrobial peptide–driven innate immune response in keratinocytes is tightly regulated by the interplay between parathyroid hormone-related protein (PTHrP) and vitamin D signaling. Under conditions of low 25(OH)D_3_ levels, keratinocytes upregulate PTHrP expression, which activates parathyroid hormone receptor type 1. This activation synergistically enhances the expression of the antimicrobial peptide cathelicidin through demethylation and activation of the VDREs thereby strengthening the skin’s antimicrobial defense (Errazahi et al. 2004; Sharpe et al. 1998; Muehleisen B et al. 2012). Furthermore, vitamin D3 modulates adaptive immunity by suppressing pathogenic Th17 cell differentiation. This occurs via downregulation of retinoic acid receptor α (RARα), a nuclear receptor critical for RORγt-mediated IL17A transcription and Th17 lineage commitment (Agak et al. 2014; Iwata et al. 2003; Khalil et al. 2025). Experimental evidence suggests that vitamin D3 directly inhibits IL-17 gene transcription, thereby obstructing the maturation of IL-17-secreting T cells and attenuating a mixed inflammatory infiltrate of lymphocytes and neutrophils (Abdel-Wahab et al. 2022).It is also important to note that polymorphisms in the VDR gene may increase the risk of acne vulgaris. In patients with acne vulgaris, genetic variations in the VDR gene are significantly associated with reduced serum 25-hydroxyvitamin D3 (25(OH)D3) levels. Specifically, a lower frequency of the VDR ApaI A allele (rs7975232) and the AATT combined genotype (a haplotype integrating ApaI and TaqI polymorphisms), alongside a higher prevalence of the TaqI tt genotype (rs731236) and t allele, correlates with diminished 25(OH)D3 concentrations compared to healthy controls (Swelam et al. 2019). These polymorphisms may impair VDR-mediated transcriptional activity or ligand binding, exacerbating vitamin D deficiency and its downstream effects on sebocyte regulation, inflammation, and microbial defense in acne pathogenesis.

### Alopecia areata

Alopecia areata (AA) is an immune-mediated dermatologic disorder characterized by cytotoxic T cell–driven attacks on hair follicle antigens, resulting in non-scarring, patchy hair loss primarily affecting the scalp, eyebrows, and/or other hair-bearing regions (MacLean and Tidman 2013; Islam et al. 2015; Kumar et al. 2025; Mostaghimi et al. 2025). Notably, AA patients demonstrate significantly lower serum 25-hydroxyvitamin D3 (25(OH)D3) levels compared to healthy controls, coupled with diminished VDR expression in lesional hair follicle keratinocytes and dermal papilla cells (Chen et al. 2007; Mahamid et al. 2014). This hypovitaminosis D and impaired VDR signaling may exacerbate immune dysregulation by reducing follicular immune privilege maintenance and disrupting hair cycle checkpoint control, positioning vitamin D supplementation as a potential adjunctive immunomodulatory strategy in AA management.

Topical calcipotriol has been reported to be successful in treating AA. Both a three-month topical calcipotriol treatment and narrowband UVB phototherapy (NB-UVB) have demonstrated efficacy in managing AA. Improvements in both the Severity of Alopecia Tool (SALT) score and serum vitamin D3 levels have been observed (Taieb et al. 2019). Notably, post-treatment vitamin D3 levels did not differ significantly between patients treated with topical calcipotriol and those undergoing narrowband ultraviolet B (NB-UVB) phototherapy. This finding suggests that calcipotriol may effectively replicate the UVB-induced vitamin D synthesis pathway typically mediated by epidermal keratinocytes, circumventing the need for direct UVB exposure to achieve comparable vitamin D bioavailability. Additionally, a case report highlights the beneficial effects of vitamin D3 and its derivatives, including calcitriol and paricalcitol, in AA patients. However, the therapeutic durability of these interventions remains limited, with disease recurrence observed in subsets of patients despite initial clinical improvement. This underscores the transient nature of immunomodulatory effects and highlights the persistence of underlying immune dysregulation in alopecia areata pathogenesis. Sustained remission may require long-term maintenance therapy or combinatorial strategies targeting upstream inflammatory drivers (Papadimitriou et al. 2021). One study demonstrated that a maternal vitamin D-deficient/low-calcium diet can lead to transient non-cicatricial alopecia in calbindin-D9k knockout mice due to impaired postnatal hair follicle cycling (Mady et al. 2016). Calbindin acts as a calcium-binding protein that helps regulations of intracellular calcium levels in immune cells and neuronal excitability regulation (Christakos and Liu 2004; Kim et al. 2021). Besides, the vitamin D-mediated neuronal protection and calcium homeostasis in immune cells. The crosstalk between VDR expression and AA is tightly innervated with each other (Cerman et al. 2015).The mutation of VDR expression has been observed in patients with AA and alopecia universalis (AU) (Duker and Brown 1988). VDR also is a ligand-activated transcription factor, interacts with the Wnt/β-catenin pathway, a central regulator of hair follicle biology. This pathway governs critical processes in outer root sheath cells, hair matrix cells, and dermal papilla cells, including hair morphogenesis, follicle regeneration, and cyclical hair growth—mechanisms whose dysregulation is implicated in AA pathogenesis (Gerkowicz et al. 2017; Wang et al. 2023; Shin 2022). Notably, VDR deficiency disrupts Wnt/β-catenin signaling, reducing cytoplasmic and nuclear β-catenin accumulation and downregulating Wnt target genes (e.g., LEF1, AXIN2) essential for hair follicle maintenance (2025; Kise et al. 2025). Experimental inhibition of VDR via small interfering RNA (siRNA) or Dickkopf-1 (DKK1), a Wnt antagonist, diminishes both VDR and β-catenin expression in dermal papilla cells, further impairing pro-growth signaling (Lim et al. 2014). These findings highlight the synergistic role of VDR and Wnt/β-catenin in preserving hair follicle integrity, with disruptions contributing to AA’s hallmark non-scarring hair loss. Future studies should delineate the molecular crosstalk between VDR and Wnt/β-catenin during hair cycle dysregulation in AA, while clinical trials are needed to evaluate whether vitamin D supplementation restores this axis, offering a mechanistic basis for therapeutic intervention.

### Systemic sclerosis (Scleroderma)

Systemic sclerosis (SSc) is marked by multisystem fibrosis, most prominently involving the skin, though often progressing to visceral organs. SSc is categorized into two primary subtypes: localized scleroderma, confined to cutaneous regions, and systemic sclerosis, which manifests with widespread organ involvement (Volkmann et al. 2023; Hinchcliff et al. 2025; Tambaro et al. 2025). Despite its rarity, SSc demands multidisciplinary management and specialized clinical expertise due to its propensity for life-threatening complications, including pulmonary arterial hypertension and end-stage organ fibrosis (Bournia et al. 2021; Perelas et al. 2020). These devastating sequelae underscore the critical imperative for advancing targeted therapeutics to mitigate disease progression.

The association between circulating 25-hydroxyvitamin D (25(OH)D) levels and systemic sclerosis (SSc) phenotypes remains inconsistent across studies. While some reports document persistent hypovitaminosis D in SSc patients, even among those receiving cholecalciferol supplementation (Groseanu et al. 2016; Runowska et al. 2021; Corrado et al. 2015; Hax et al. 2020), others identify an inverse correlation between 25(OH)D concentrations and validated disease activity indices, such as the European Scleroderma Study Group Activity Index (ESSGAI) (Atteritano et al. 2016; Gupta et al. 2018; Vacca et al. 2009). Conversely, subsequent analyses have failed to show a correlation between circulating vitamin D levels and key inflammatory cytokines (Feki et al. 2023). These discrepancies may reflect heterogeneity in SSc subtypes (e.g., limited vs. diffuse cutaneous involvement), variability in supplementation protocols, or confounding factors such as sun exposure and renal function. Many foods today, such as milk, contain varying amounts of vitamin D. Differences in vitamin D intake during the study could have influenced the outcomes. Dietary intake of vitamin D-rich foods (e.g., fatty fish, fortified dairy) can mitigate hypovitaminosis D in populations with limited solar UVB exposure, such as residents of northern latitudes or individuals with predominantly indoor lifestyles (Amrein et al. 2020). However, existing studies often fail to account for dietary confounders, including calcium intake, phytate-rich foods, or fat malabsorption syndromes, which may influence vitamin D bioavailability and confound observed associations.

Seasonal variations in vitamin D synthesis are paralleled by photoperiod-driven fluctuations in sex steroid hormones. For instance, healthy young males exhibit summertime elevations in serum testosterone and luteinizing hormone (LH), correlating with peak serum 25-hydroxyvitamin D (25(OH)D) levels (Andersson et al. 2003). These circannual rhythms suggest shared regulatory mechanisms between vitamin D metabolism and endocrine signaling.Notably, a Thai cohort study of systemic sclerosis (SSc) patients reported seasonal patterns in healthcare utilization, with hospital admission rates highest during the rainy season (low UVB availability) and lowest in the hot season (Foocharoen et al. 2020). While this aligns with hypothesized links between vitamin D deficiency and SSc exacerbations, the study did not measure serum 25(OH)D levels, precluding direct assessment of vitamin D’s role in observed seasonal morbidity. Future studies should integrate longitudinal 25(OH)D measurements with clinical and environmental data to disentangle these interactions.

VDR mRNA expression exhibits seasonal variation, reaching nadir levels in late winter and early spring, which inversely correlates with mRNA and serum levels of inflammatory mediators in SSc (Dal-Bekar et al. 2023). Paradoxically, comparative analyses reveal no significant differences in these inflammatory biomarkers between SSc patients and healthy controls, suggesting that VDR downregulation may reflect a secondary phenomenon rather than a primary driver of SSc pathogenesis (Jacquerie et al. 2021; Zerr et al. 2015). While hypovitaminosis D is prevalent in SSc cohorts, its etiopathogenic role remains inconclusive due to conflicting observational data and heterogeneity in study designs. Future investigations should adopt longitudinal, multifactorial frameworks integrating genetic (e.g., VDR polymorphisms), environmental (e.g., UVB exposure, latitude), and immune-fibrotic biomarkers to disentangle vitamin D’s contributory role from epiphenomenal associations in SSc.

Significant advancements have been made in elucidating the pathobiology of SSc, a disorder marked by immune dysregulation and progressive fibrosis. Aberrant innate and adaptive immune responses drive autoantibody production and cell-mediated autoimmunity, with notable disruptions in T cell subset homeostasis—particularly in Th17 cell populations. Patients with SSc exhibit elevated Th17 cell frequencies compared to healthy controls, as demonstrated by heightened expression of RORγt (the master transcriptional regulator of Th17 differentiation), increased IL-17A mRNA levels, and elevated proinflammatory cytokines (IL-1β, IL-6, and TNF-α). These findings are further corroborated by a higher proportion of CD4^+^IL-17A^+^ T cells in SSc patient cohorts (Wei et al. 2022; Moon et al. 2021; Gabsi et al. 2019). Accumulating studies now implicate chronically activated fibroblasts as key mediators of dysregulated collagen biosynthesis and ECM deposition across dermal and internal organ microenvironments. This dysregulated ECM remodeling underlies the pathological skin thickening and multi-organ fibrosis hallmark of SSc, perpetuating its characteristic tissue stiffening and functional impairment (Asano 2020; Truchetet et al. 2023; Dobrota et al. 2024; Lozinski et al. 2024).

**Fig. 3:**
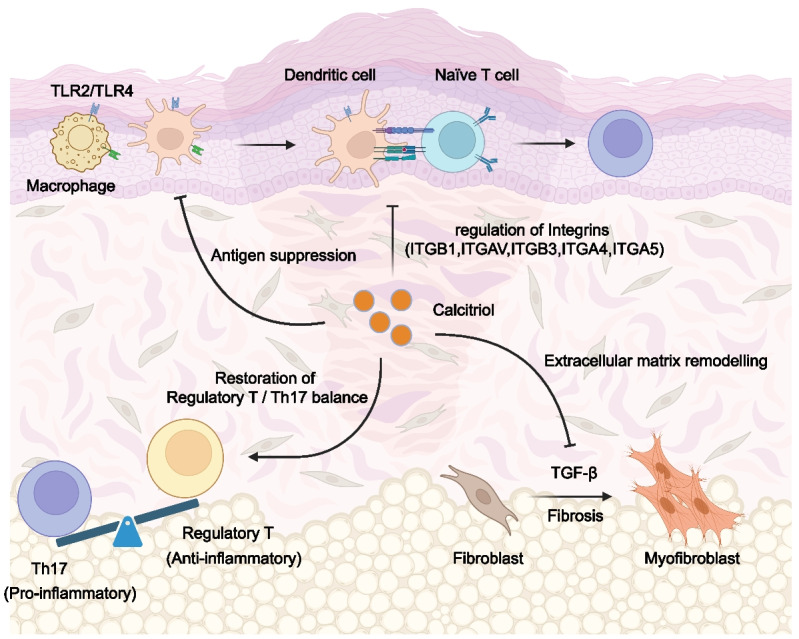
Mechanisms and consequences of calcitriol in Scleroderma. Scleroderma encompasses a complex spectrum of disorders characterized by immune dysregulation and fibrosis. Calcitriol demonstrates potential therapeutic effects by modulating both immune responses and fibrotic processes.In terms of immune modulation, calcitriol reduces the antigenpresenting capability of macrophages and dendritic cells. It also diminishes the efficiency and specificity of T cell activation by suppressing various integrins involved in the interactions between T lymphocytes and antigen-presenting cells. This leads to a restoration of the balance between regulatory T cells and Th17 cells, primarily by promoting regulatory T cell populations and fostering an anti-inflammatory microenvironment.On the fibrotic front, calcitriol exerts its effects by reshaping the extracellular matrix, notably through the inhibition of TGF-β-mediated fibrosis.ITGA4, integrin subunit alpha 4; ITGA5, integrin subunit alpha 5; ITGAV, integrin subunit alpha V; ITGB1, integrin beta-1; ITGB3, integrin subunit beta 3; TGF-β, transforming growth factor beta; Th17, type 17 T helper cells; TLR2, toll-like receptor 2; TLR4, toll-like receptor 4

Cholecalciferol administration increases Treg levels in SSc patients as shown in Fig. 3. Increased populations of Tregs may contribute to suppression of T cell proliferation (Liberto et al. 2020; Zhang et al. 2024),resulting in the inhibitory effects on both IL-17A and pro-fibrotic cytokines (Corrado et al. 2022).Notably, this inhibitory maybe due to 1,25(OH)_2_D_3_ suppressing the TGF-β1 function in Th17 cell differentiation. TGF-β1 synergizes with IL-6 to orchestrate Th17 cell differentiation from naive CD4 + T cells by inducing the master transcriptional regulator RORγt,a critical driver of SSc pathogenesis (Zhang 2018; Choi et al. 2021). Vitamin D modulates the maturation and activation of macrophages and dendritic cells, shifting their functional phenotype toward immunosuppressive or regulatory states. This immunomodulatory effect attenuates their capacity to function as potent antigen-presenting cells (APCs), thereby influencing immune homeostasis (Piemonti et al. 2000) (Fig. 3). Upon TLR activation, macrophages and dendritic cells upregulate surface molecules—including MHC-II, CD80/CD86, and CD40—and secrete cytokines, collectively enabling efficient antigen presentation to naïve T cells and driving Th17 cell differentiation. Intriguingly, vitamin D3 suppresses the surface expression of TLR2 and TLR4 in monocytes, macrophages, and dendritic cells, suggesting a regulatory mechanism to temper TLR-mediated pro-inflammatory signaling and downstream Th17 polarization (Sadeghi et al. 2006; Gambhir et al. 2011; Gheitanchi et al. 2025; Vafaeian et al. 2025).

Notably, the non-calcemic vitamin D analog 17,20S(OH)_2_pD attenuates dermal fibrosis progression in a bleomycin-induced SSc model, as demonstrated by reductions in collagen production, dermal thickening, and extracellular matrix deposition, alongside diminished subcutaneous adipose tissue loss. Mechanistically, this antifibrotic effect is mediated through suppression of the TGF-β1/Smad signaling axis, highlighting its therapeutic potential to disrupt profibrotic pathways central to SSc pathogenesis (Brown Lobbins et al. 2021). The antifibrotic effects of 1,25(OH)_2_D_3_ on cytokine production—particularly pro-fibrotic mediators—are observed in vitro only at supraphysiological concentrations, which far exceed physiological levels. While this translational gap complicates the extrapolation of findings to clinical vitamin D supplementation in humans, the mechanistic insights derived from these studies retain pathophysiological relevance, particularly in elucidating vitamin D’s capacity to modulate profibrotic pathways such as TGF-β signaling.

The possible mechanisms by which VDR deficiency contributes to the development of SSc are multifaceted. SSc patients have significantly lower VDR expression compared to healthy individuals (Dal-Bekar et al. 2023).VDR deficiency is strongly correlated with the modulation of integrins, including ITGB1, ITGAV, ITGB3, ITGA4, and ITGA5 (Li et al. 2020). These integrins play an important role for chemokines signal transduction as well as the proliferation, migration and differentiation in inflamed tissues (Wu et al. 2020; Taketomi et al. 2024).

Additionally, knockdown of VDR enhances the sensitivity of fibroblasts to TGF-β, resulting in aberrant fibroblast activation in SSc and fibrosis (Zerr et al. 2015; Trinh-Minh et al. 2024).In addition to regulating integrin proteins for immune cell functions, VDR is also linked to reducing ROS generation and suppressing ROS-dependent pathways by restoring mitochondrial ATP production, complex V activity, as well as protecting the integrity of mitochondria-associated membranes (Chen et al. 2024). Notably, the reduction in oxidative DNA damage product 8-oxo-dG levels after vitamin D administration in scleroderma patients with lung, joint, and gastrointestinal involvement pinpoints the efficacy of vitamin D in individuals with organ-specific manifestations of the disease (Dal-Bekar et al. 2023).

### Chronic spontaneous urticaria (CSU)

Chronic spontaneous urticaria (CSU) is categorized as an allergic skin disorder, characterized by the sudden onset of hives (urticaria) and/or angioedema. It can affect various areas of the skin, including the eyes, lips, and throat. However, despite its prevalence and the availability of effective anti-IgE therapies(eg. Omalizumab), CSU is often associated with disease recurrence (2025; Kucharczyk et al. 2024), which can lead to significant emotional distress and further diminish patients’ quality of life (Cetinkaya et al. 2019; Caffarelli et al. 2019; Zuberbier et al. 2014). Strikingly, a positive correlation has been observed between urticaria and vitamin D deficiency (Liu et al. 2024). Patients with CSU often exhibit lower serum levels of 25(OH)D (Vurgun et al. 2020; Thorp et al. 2010). This deficiency appears to be independent of age, sex, and disease duration in CSU patients (Li et al. 2021; Tsai and Huang 2018; Mohamed et al. 2022; Rather et al. 2018).

In clinical trials, two different doses of vitamin D3 treatment (Low: 4200 IU/week; high: 28,000 IU/week) over 12 weeks have been examined for CSU patients. Both doses demonstrate therapeutic effects by week 6, as evidenced by reductions in total urticaria severity scores(USS) and increases in quality of life scores (2023). Interestingly, significant increases in serum 25-hydroxyvitamin D levels are only observed at week 12 in the high-dose group (2023). Administering high doses of vitamin D3 may not be essential for managing skin diseases and could help mitigate adverse effects associated with high doses, such as neurological symptoms, pancreatitis, and hypercalcemia (Kohli et al. 2023; Galior et al. 2018).

A separate randomized trial administering daily 0.25 µg alfacalcidol (1α-hydroxycholecalciferol), an active vitamin D analog, over 12 weeks demonstrated significant elevation of serum 25-hydroxyvitamin D [25(OH)D] levels compared to placebo, alongside marked attenuation of inflammatory biomarkers (Mohamed et al. 2022). Additionally, administering high-dose vitamin D (60,000 IU/week) for 4–12 weeks shows potential in decreasing disease activity in some CSU patients (Li et al. 2021; Tuchinda et al. 2018).Moreover, the addition of a standardized triple-drug regimen—comprising cetirizine, ranitidine, and montelukast—alongside high-dose vitamin D3 (4,000 IU daily for 12 weeks) may offer a safe and potentially effective immunomodulatory option for patients with chronic urticaria. Clinical studies indicate that vitamin D3 supplementation, as an adjunct to standard therapies, demonstrates efficacy in enhancing sleep quality, alleviating pruritus, and reducing Urticaria Severity Score (USS) values in CSU (Rorie et al. 2014). These findings demonstrate its capacity as a valuable adjunct therapy for enhancing clinical outcomes in patients with CSU. Furthermore, vitamin D3 exhibits synergistic efficacy when co-administered with anti-asthmatic medications (2022), likely attributable to overlapping mechanisms of action.

The working mechanism behind the therapeutic effect of vitamin D3 on CSU involves several key processes. Urticaria is thought to be a consequence of mast cell activation. Activated mast cells provoke vasodilation, plasma extravasation, and sensory nerve hypersensitivity, which recruits more immune cells, such as neutrophils and additional mast cells, leading to the release of lipid mediators like prostaglandins and platelet-activating factor (Zuberbier and Maurer 2007). VEGF production in mast cells occurs in an IgE-dependent manner via the PI3K/Akt/p38 MAPK/HIF-1α axis. Importantly, 25(OH)D3 attenuates the expression of VEGF by suppressing this signaling pathway (Zhao et al. 2020). In addition to mast cells, the pathogenesis of CSU involves an inflammatory infiltrate with the interplay of multiple effector cells, including T cells, basophils, and eosinophils (Altrichter et al. 2020).Vitamin D3 significantly decreases serum levels of IL-6, hsCRP, and TNFα (Mohamed et al. 2022).This effect is possibly mediated by vitamin D binding proteins (DBP), which have immunomodulatory effects in the human body without affecting free vitamin D concentration (Bikle and Schwartz 2019).VDBP may be compensatory increased due to vitamin D deficiency in CSU to reduce inflammation and disease activity. VDR gene single nucleotide polymorphisms (TaqI and FokI) has been linked to the pathogenesis of CSU. Individuals who carry GCCA haplotype show a decrease in vitamin D levels with the G allele of TaqI and A allele of FokI gene SNPs (2022).

Apart from the compensatory mechanism of VDBP, vitamin D3 also modulates the gut microbiome to reduce the severity of CSU. Vitamin D possibly increases fecal genera such as *Prevotella* 9, *Escherichia–Shigella*, and *Klebsiella*, while notably reducing *Bacteroides*, *Faecalibacterium*, and *Agathobacter*, resulting in the restoration of type I interferon homeostasis (Yang et al. 2024). However, the mechanisms by which gut microbiota dysbiosis contributes to skin inflammatory diseases remain inadequately understood. Gut microbiota can alter type I interferon levels, either by secreting metabolites or by directly affecting mast cell development (a major source of type I interferon) in the context of other diseases (Shimbori et al. 2022; Zuani et al. 2018). Additionally, the causal relationship between skin and gut microbiota needs to be clarified in greater detail before considering its clinical application.

## Conclusions

Vitamin D deficiency, a widespread global condition, has long been debated for its potential link to various skin diseases. This review consolidates extensive evidence on the roles of vitamin D in the etiology, development, and progression of skin conditions. Preclinical studies convincingly demonstrate the impact of the vitamin D/VDR axis on these processes. However, the large inconsistencies in study design, outcome assessments, and conclusions of current clinical studies limit definitive conclusions. Currently, vitamin D analogues are used as anti-inflammatory agents and serve as adjunctive treatment for psoriasis. However, their effectiveness in treating other skin diseases remains uncertain.

The increasing global burden of inflammatory skin diseases, such as psoriasis and eczema, attributed to lifestyle, diet, and comorbidities, has prompted researchers to investigate vitamin D supplementation as a preventive or adjunctive treatment strategy. The immunomodulatory actions of vitamin D, its non-toxicity, and low cost render it an appealing choice, with research pointing toward its role in enhancing the skin barrier function and influencing inflammatory pathways. Yet genetic variation within the vitamin D receptor (VDR)—notably polymorphisms like the TaqI variant allele and FokI FF genotype—significantly influences subject responsiveness to supplementation (Usategui-Martin et al. 2022), which can perhaps explain discordant findings in earlier studies. This kind of genetic variation highlights the need for personalized approaches in future clinical trials, in which stratification of subjects according to VDR genotypes would identify subgroups most likely to benefit, in line with precepts of precision medicine. To completely unlock vitamin D's potential, greater mechanistic insight into its influence on cytokine networks, neuroprotection mechanisms, and immune regulation is required, as well as rigorous investigations of dosing variability, long-term safety, and interactions with lifestyle factors like sun exposure. Experimental findings will be translated into actionable therapies by spanning basic research, i.e., molecular studies of VDR signaling, and clinical trials. While vitamin D is a relatively safe treatment, maximizing its therapeutic promise will require an acceptance of genetic diversity and an interdisciplinary effort to ensure robust, reproducible findings. Ultimately, the integration of genetic screening and individualized strategies has the potential to make vitamin D a cornerstone of dermatologic therapy, providing targeted benefit to risk groups and advancing the overall movement toward individualized, preventive medicine.

## Data Availability

No datasets were generated or analysed during the current study.
